# Guanylate-Binding Protein 1 as a Potential Predictor of Immunotherapy: A Pan-Cancer Analysis

**DOI:** 10.3389/fgene.2022.820135

**Published:** 2022-02-10

**Authors:** Yaqi Zhao, Jie Wu, Lan Li, Huibo Zhang, Haohan Zhang, Jing Li, Hao Zhong, Tianyu Lei, Yan Jin, Bin Xu, Qibin Song

**Affiliations:** ^1^ Cancer Center, Renmin Hospital of Wuhan University, Wuhan, China; ^2^ Department of Bioinformatics, Wissenschaftszentrum Weihenstephan, Technical University of Munich, Freising, Germany

**Keywords:** GBP1, pan-cancer, predictive biomarkers, immunotherapy, prognosis, immune cell infiltration

## Abstract

**Background:** Mainstream application of cancer immunotherapy is hampered by the low response rate of most cancer patients. A novel immunotherapeutic target or a biomarker predicting response to immunotherapy needs to be developed. Guanylate-binding protein 1 (GBP1) is an interferon (IFN)-inducible guanosine triphosphatases (GTPases) involving inflammation and infection. However, the immunological effects of GBP1 in pan-cancer patients are still obscure.

**Methods:** Using large-scale public data, we delineated the landscape of GBP1 across 33 cancer types. The correlation between GBP1 expression or mutation and immune cell infiltration was estimated by ESTIMATE, TIMER, xCell, and quanTIseq algorithms. GBP1-related genes and proteins were subjected to function enrichment analysis. Clustering analysis explored the relationship between GBP1 expression and anti-tumor immune phenotypes. We assessed the patient’s response to immunotherapy using the tumor immune dysfunction and exclusion (TIDE) score and immunophenoscore (IPS). Furthermore, we validated the predictive power of GBP1 expression in four independent immunotherapy cohorts.

**Results:** GBP1 was differentially expressed in tumors and normal tissues in multiple cancer types. Distinct correlations existed between GBP1 expression and prognosis in cancer patients. GBP1 expression and mutation were positively associated with immune cell infiltration. Function enrichment analysis showed that GBP1-related genes were enriched in immune-related pathways. Positive correlations were also observed between GBP1 expression and the expression of immune checkpoints, as well as tumor mutation burden (TMB). Pan-cancer patients with higher GBP1 expression were more inclined to display “hot” anti-tumor immune phenotypes and had lower TIDE scores and higher immunophenoscore, suggesting that these patients had better responses to immunotherapy. Patients with higher GBP1 expression exhibited improved overall survival and clinical benefits in immunotherapy cohorts, including the Gide et al. cohort [area under the curve (AUC): 0.813], the IMvigor210 cohort (AUC: 0.607), the Lauss et al. cohort (AUC: 0.740), and the Kim et al. cohort (AUC: 0.793).

**Conclusion:** This study provides comprehensive insights into the role of GBP1 in a pan-cancer manner. We identify GBP1 expression as a predictive biomarker for immunotherapy, potentially enabling more precise and personalized immunotherapeutic strategies in the future.

## Introduction

*Cancer* immunotherapy aiming at reactivating the anti-tumor immune response has emerged as a new therapeutic pillar of oncology (Waldman et al., 2020). It includes lymphocyte-promoting cytokines, engineered T cells, cancer vaccines, and immune checkpoint inhibitors (ICIs) that can boost immune cell activity by blocking immune checkpoint targets (Waldman et al., 2020). Tumor regression and durable responses were observed in a proportion of patients treated with ICIs, and long-lasting tumor-specific immunological memory remained appreciable even after cessation of treatment (Waldman et al., 2020). Despite the impressive clinical outcomes, only approximately 20% of cancer patients can benefit from immunotherapy, and some of them eventually relapse after a period of response (Sharma et al., 2017). To identify the patients with higher response rates, researchers have proposed several predictive biomarkers such as PD-L1 expression levels (Hui et al., 2017) and tumor mutation burden (TMB) (Osipov et al., 2020). However, none of these markers has been fully validated yet; thus, we wished to find a novel biomarker for immunotherapy.

Guanylate-binding proteins (GBPs) belong to a family of interferon (IFN)-inducible guanosine triphosphatases (GTPases) (Tretina et al., 2019). To date, the human GBPs consist of seven family members, and several of them play critical roles in inflammation and infection processes (Tretina et al., 2019). Guanylate-binding protein 1 (GBP1), the best-characterized member of GBPs, comprises a long C-terminus of parallel α-helices mediating the inhibition of cell proliferation and a globular N-terminus with gtpase activity that can control endothelial cell invasion and angiogenesis *via* repression of matrix metalloproteinase-1 (MMP-1) expression (Guenzi et al., 2003). GBP1 activation provoked by IFN and other cytokine stimulation involves cellular responses to infection and inflammation by preventing the proliferation of infected, endothelial, and epithelial cells and protecting cells from apoptosis (Honkala et al., 2019). In the context of cancer, the effects of GBP1 appear to be highly complex. High GBP1 expression inhibited tumor cell proliferation in breast and colorectal cancers (Lipnik et al., 2010; Britzen-Laurent et al., 2013), but it was strongly associated with disease progression and paclitaxel resistance in ovarian cancer and glioblastoma (Li et al., 2011; Wadi et al., 2016). It is noteworthy that GBP1 expression has been reported to correlate with the presence of an IFN-γ-dominated T helper type 1 (Th1) immune response in colorectal carcinoma (Naschberger et al., 2008). Our previous study constructed a risk model developed based on eight genes, including GBP1, to predict prognosis and associate with tumor immunity of patients with lung adenocarcinoma (LUAD) (Wu et al., 2021). Since the effects of GBP1 across different cancers remain elusive and the potential role of GBP1 in anti-tumor immune response has been represented in the previous studies, a comprehensive pan-cancer analysis is urgently needed to explore the possibility of GBP1 being a biomarker for immunotherapy.

In this study, we used the RNA-seq and clinical data from The Cancer Genome Atlas (TCGA) database and four immunotherapy cohorts to evaluate the association between GBP1 expression and the efficacy of immunotherapy in pan-cancer patients. The results suggested that GBP1 expression might be a predictive biomarker for pan-cancer patients receiving immunotherapy. In addition, GBP1 expression was strongly associated with elevated immune cell infiltration, expression of immune checkpoints, activated anti-tumor immunity, and improved overall survival (OS) for patients treated with immunotherapy. A workflow of the whole study is provided in Supplementary Figure S1.

## Methods

### GBP1 Expression in Pan-Cancer

The differential expression of GBP1 between tumor tissues across 33 cancer types in the TCGA database and adjacent normal tissues was obtained by Tumor Immune Estimation Resource, version 2.0 (TIMER2.0), webserver (http://timer.comp-genomics.org/) (Li et al., 2020). In terms of several cancer types without paired normal tissues, we replenished the normal samples from the GTEx database using Gene Expression Profile Interactive Analysis, version 2 (GEPIA2) (http://gepia2.cancer-pku.cn/), web server (Tang et al., 2019). Proteomic expression profiles of GBP1 protein were derived from the Clinical Proteomic Tumor Analysis Consortium (CPTAC) dataset using the UALCAN portal (http://ualcan.path.uab.edu/) (Chen et al., 2019). Finally, we used the Human Protein Atlas (HPA) database (Pontén et al., 2008) to verify the distribution of GBP1 protein in tumor and normal tissues by immunohistochemistry (IHC). Direct links to the IHC image are provided in Supplementary Table S1.

### Survival Analysis of GBP1

GEPIA2 was used to analyze OS and disease-free survival (DFS) across 33 cancer types. The patients were assigned to high and low GBP1 expression groups by the median GBP1 expression, and two survival heat maps showed the OS and DFS significance maps of GBP1, respectively.

### GBP1 Expression Was Associated With Immune Cell Infiltration in Tumors

RNA-seq data of TCGA Pan-Cancer (PANCAN) cohort were downloaded from the University of California Santa Cruz Xena (UCSC Xena) browser (https://xenabrowser.net/). Protein coding genes were annotated by Ensembl (http://www.ensembl.org). The gene expression levels were normalized to transcripts per kilobase million (TPM) and transformed as log2 (TPM + 1) for downstream analysis. A total number of 33 cancer types and 9,094 cancer patients were included in this study. Estimation of STromal and Immune cells in MAlignant Tumor tissues using Expression data (ESTIMATE) algorithm can infer the fraction of immune/stromal cells and tumor purity by taking advantage of the transcriptional profiles (Yoshihara et al., 2013). Immune and stromal scores calculated by the ESTIMATE algorithm reflected the infiltration of immune and stromal cells in tumor tissues, respectively. ESTIMATE score was negatively related to tumor purity that was defined as the proportion of tumor cells in the admixture. TIMER, xCell (Aran et al., 2017), and quanTIseq (Finotello et al., 2019) algorithms were applied to further explore the correlations of GBP1 expression and tumor-infiltrating immune cells.

### GBP1 Alteration in Pan-Cancer

The alteration frequency, mutation types, and copy number alteration (CNA) across TCGA cancer types and the survival plots of patients with or without GBP1 alteration were obtained on the cBioPortal web (http://www.cbioportal.org/) (Cerami et al., 2012). The mutated site information about GBP1 can be displayed in the schematic diagram of the protein structure or the three-dimensional (3D) structure. Then, we compared the levels of infiltrating immune cells between patients with wild-type (WT) GBP1 and those with mutated GBP1 by TIMER2.0.

### GBP1-Related Gene and Protein Enrichment Analysis

For gene annotation enrichment analysis in pan-cancer patients, we identified GBP1-related genes whose expression levels were correlated with GBP1 (absolute value of Spearman correlation coefficients >0.4, *p *< 0.05). A false discovery rate (FDR) value (the adjusted *p*-value calculated using the Benjamini–Hochberg method) <0.05 indicated a significant difference for Gene Ontology (GO) terms and Kyoto Encyclopedia of Genes and Genomes (KEGG) pathways.

Moreover, we ranked genes of each cancer type according to the Spearman correlation coefficients, respectively. These gene lists were used for Gene Set Enrichment Analysis (GSEA) based on GO, KEGG, and Reactome pathway databases. A *p*-value <0.05, FDR *q*-value <0.05, and absolute value of normalized enrichment scores (NES) >1.5 were considered as significant.

The protein–protein interaction (PPI) network of GBP1 protein was constructed using the Search Tool for the Retrieval of Interacting Genes (STRING) (https://www.string-db.org/) (Szklarczyk et al., 2019). A confidence score >0.4 was set as significant.

### Analysis of GBP1 Expression and Immunotherapy

#### The Association of GBP1 Expression With the Expression of Immune Checkpoints and Tumor Mutational Burden

Nine immune checkpoints included programmed cell death 1 (PD-1, PDCD1), programmed cell death ligand 1 (PD-L1, CD274), cytotoxic T-lymphocyte-associated protein 4 (CTLA4), lymphocyte activation gene-3 (LAG3), T cell immunoglobulin and ITIM domain (TIGIT), mucin-domain containing-3 (TIM-3, HAVCR2), V-domain immunoglobulin suppressor of T cell activation (VISTA, C10orf54), B7 homolog 3 protein (B7-H3, CD276), and B and T cell lymphocyte attenuator (BTLA). TMB was defined as the total number of non-synonymous mutations per million bases of genome examined, and the R package “TCGAmutations” was used to calculate it (Ellrott et al., 2018).

#### The Association of GBP1 Expression With Anti-Tumor Immune Phenotype

To investigate the links between GBP1 expression and immune phenotype, we manually selected 14 immune-related gene sets covering both innate and adaptive immune responses against tumors from Molecular Signatures database (MsigDB, https://www.gsea-msigdb.org/gsea/msigdb/genesets.jsp) and evaluated the immune status of each patient by Gene Set Variation Analysis (GSVA) from R package “GSVA” (Hänzelmann et al., 2013). Details of the gene sets are provided in Supplementary Table S2. GSVA enrichment scores were used to cluster the patients through agglomerative hierarchical clustering. Three categories were defined as “high immune cluster (HIC),” “medium immune cluster (MIC),” and “low immune cluster (LIC).”

#### Prediction of Therapeutic Benefits in Patients With Different Expression Levels of GBP1

The patient’s response to immunotherapy was estimated by the tumor immune dysfunction and exclusion (TIDE) score (Fu et al., 2020) and immunophenoscore (IPS) (Charoentong et al., 2017). The TIDE scores of pan-cancer patients were obtained from the TIDE web platform (http://tide.dfci.harvard.edu/). We set the threshold of the TIDE score at 0 and thus considered patients with negative TIDE scores as responders. Generally, a patient with lower TIDE score and higher IPS is speculated to respond better to immunotherapy.

#### Validation of the Predictive Capacity of GBP1 Expression in Immunotherapy

Gene expression profiles and clinical information of four independent cohorts were downloaded to validate the predictive value of GBP1 expression in immunotherapy. Four immunotherapy cohorts included the Gide et al. cohort (Gide et al., 2019), the IMvigor210 cohort (Mariathasan et al., 2018), the Lauss et al. cohort (Lauss et al., 2017), and the Kim et al. cohort (Kim et al., 2018), and the details are displayed in Table 1. According to Response Evaluation Criteria in Solid Tumors (RECIST) criteria, responders were defined as patients who had complete or partial responses (CR/PR) after immunotherapy; non-responders were those who had stable disease (SD) or progressive disease (PD). The predictive power of GBP1 expression and other biomarkers in the immunotherapy cohorts was used to construct receiver operating characteristic (ROC) curves, and the area under the curve (AUC) values were calculated.

**TABLE 1:** Details of immunotherapy cohorts.

Name	Therapy	Cancer type	Number of patients	Response evaluation criteria	Source
Gide et al. cohort (Gide et al., 2019)	Anti-PD-1 monotherapy or combined anti-PD-1 and anti-CTLA4 therapy	Melanoma	73	RECIST	http://tide.dfci.harvard.edu/download/
IMvigor210 cohort (Mariathasan et al., 2018)	Anti-PD-L1 therapy	Metastatic urothelial cancer	298	RECIST	http://research-pub.gene.com/IMvigor210CoreBiologies/
Lauss et al. cohort (Lauss et al., 2017)	Adoptive T cell therapy	Melanoma	25	RECIST	https://www.ncbi.nlm.nih.gov/geo/query/acc.cgi?acc=GSE100797
Kim et al. cohort (Kim et al., 2018)	Anti-PD-1 therapy	Metastatic gastric cancer	45	RECIST	http://tide.dfci.harvard.edu/download/

### Statistical Analysis

The Kaplan–Meier plotter was employed to generate survival curves for subgroups in each cohort. The significance of survival curves was estimated by the log-rank test. Univariate and multivariate Cox regression analyses were performed to identify prognostic factors. Comparisons were made by Wilcoxon (two groups) or Kruskal–Wallis (three groups) tests. Correlations were tested by the Pearson (*r*) or Spearman (*R*/Rho) tests when appropriate. Proportions were tested using the chi-square test or Fisher’s exact test. A two-tailed *p*-value <0.05 was considered as statistically significant. Analyses were performed with R software (version 3.6).

## Results

### GBP1 Expression in Pan-Cancer

**FIGURE 1:**
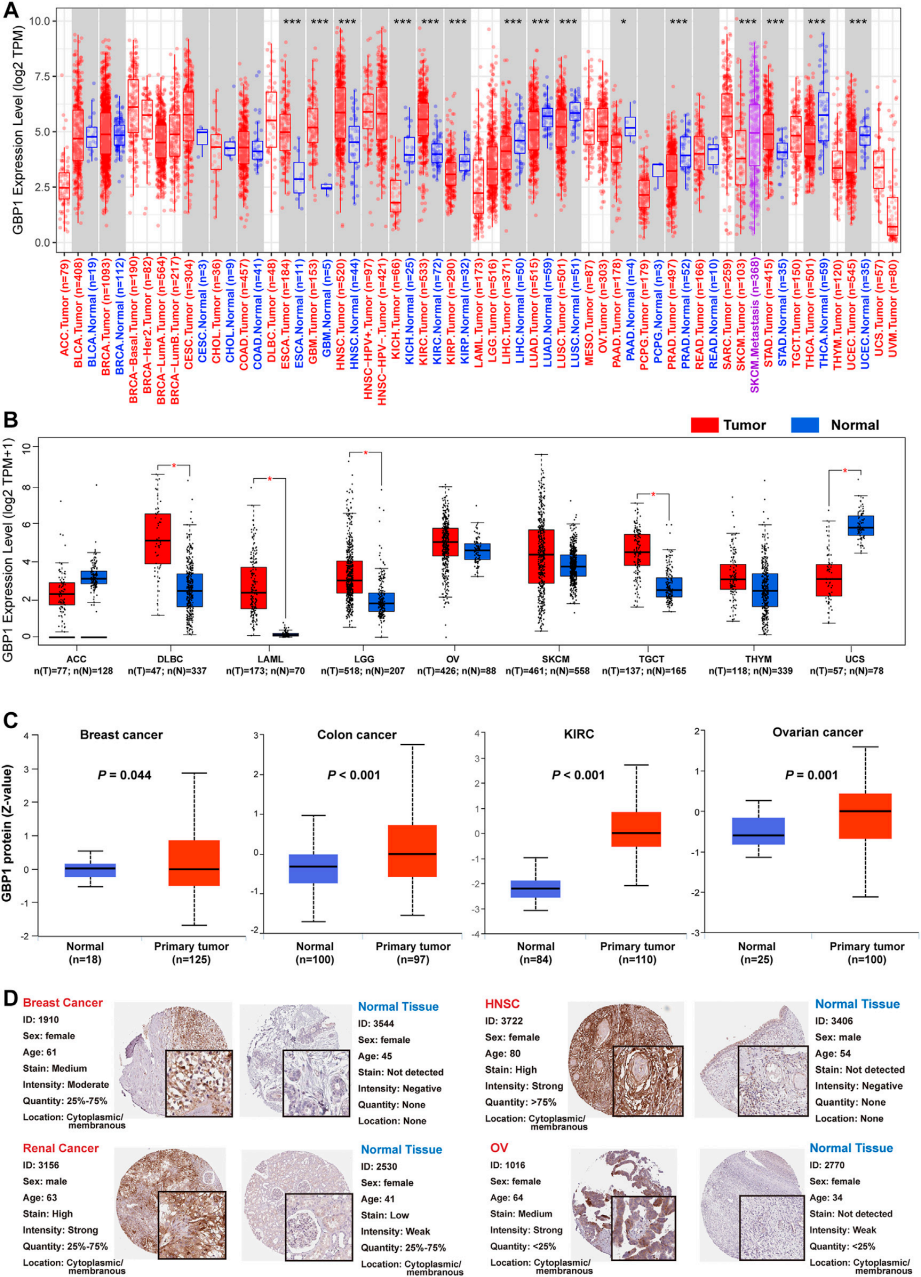
expression level of GBP1 gene and protein in different tumors and their corresponding normal tissues. **(A)** The expression level of the GBP1 gene in normal tissues and tumors across 33 TCGA cancer types or specific cancer subtypes was analyzed by TIMER2.0. *p < 0.05; **p < 0.01; ***p < 0.001. **(B)** For the cancer type of ACC, DLBC, LAML, LGG, OV, SKCM, TGCT, THYM, and UCS in the TCGA database, matched TCGA normal and GTEx data were included as control. Box plot depicts GBP1 expression in tumor and normal tissues. *p < 0.05. **(C)** The expression level of GBP1 total protein between normal tissues and primary tissues of BRCA, colon cancer, KIRC, and OV. **(D)** The differential expression of GBP1 proteins between tumor and normal tissues in the HPA database.

Among 33 cancer types of TCGA database, GBP1 showed lower expression levels in tumor samples of kidney chromophobe (KICH) (*p *< 0.001), kidney renal papillary cell carcinoma (KIRP) (*p *< 0.001), liver hepatocellular carcinoma (LIHC) (*p *< 0.001), LUAD (*p *< 0.001), lung squamous cell carcinoma (LUSC) (*p *< 0.001), prostate adenocarcinoma (PRAD) (*p *< 0.001), thyroid carcinoma (THCA) (*p *< 0.001), uterine corpus endometrial carcinoma (UCEC) (*p *< 0.001), and pancreatic adenocarcinoma (PAAD) (*p *< 0.05) compared with corresponding normal controls. In contrast, GBP1 expression was elevated in tumor samples of esophageal carcinoma (ESCA) (*p *< 0.001), glioblastoma multiforme (GBM) (*p *< 0.001), kidney renal clear cell carcinoma (KIRC) (*p *< 0.001), stomach adenocarcinoma (STAD) (*p *< 0.001), and head and neck squamous cell carcinoma (HNSC) (*p *< 0.001). Besides, the GBP1 expression was significantly upregulated in metastasis samples of skin cutaneous melanoma (SKCM) versus primary tumor samples (*p *< 0.001) (Figure 1A). After matching normal tissues from TCGA and GTEx databases, higher GBP1 expression levels were observed in tumors of diffuse large B-cell lymphoma (DLBC) (*p *< 0.05), acute myeloid (LAML) (*p *< 0.05), brain lower grade glioma (LGG) (*p *< 0.05), and testicular germ cell tumors (TGCT) (*p *< 0.05), while tumors of uterine carcinosarcoma (UCS) (*p *< 0.05) showed lower GBP1 expression compared to normal tissues (Figure 1B).

GBP1 total protein expression was higher in primary tumors, including breast cancer (*p* = 0.044), colon cancer (*p *< 0.001), KIRC (*p *< 0.001), and ovarian cancer (*p* = 0.001), than corresponding normal tissues (Figure 1C). The results of IHC showed that GBP1 protein was overexpressed in tumor tissues of breast cancer, HNSC, renal cancer, and ovarian serous cystadenocarcinoma (OV) compared with normal tissues (Figure 1D), and GBP1 protein was mainly located in the cytoplasm and membrane of tumor cells. Overexpression of GBP1 protein appeared in tumor tissues of bladder urothelial carcinoma (BLCA), cervical and endocervical cancers (CESC), cholangiocarcinoma (CHOL), lymphoma, glioma, LIHC, LUSC, PAAD, and STAD, while the converse appeared in colon adenocarcinoma (COAD) and THCA (Supplementary Figure S2).

### Survival Analysis of GBP1

High GBP1 expression was associated with poor OS prognosis for patients with KIRP (*p* = 0.0061), LGG (*p *< 0.001), thymoma (THYM) (*p* = 0.026), and uveal melanoma (UVM) (*p* = 0.0018), while high GBP1 expression conferred a better prognosis for patients with OV (*p* = 0.0038) and SKCM (*p *< 0.001) (Supplementary Figure S3A). Additionally, high GBP1 expression was related to poor DFS prognosis for KIRP (*p* = 0.001) and LGG (*p *< 0.001) (Supplementary Figure S3B).

### GBP1 Expression Was Associated With Immune Cell Infiltration in Tumors

**FIGURE 2:**
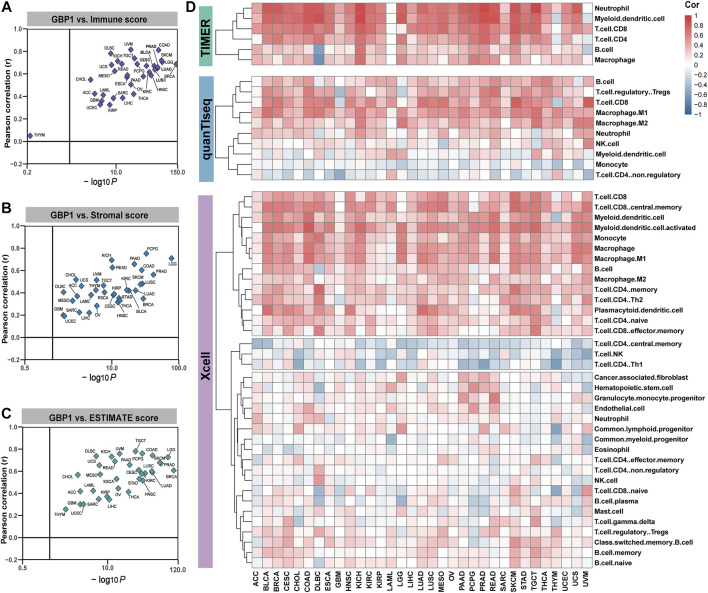
correlation between GBP1 expression and immune cell infiltration. The correlation of GBP1 expression with immune **(A)**, stromal **(B)**, and ESTIMATE **(C)** scores across 33 cancer types. **(D)** The correlation between GBP1 expression and the infiltration level of immune cells across 33 cancer types calculated by TIMER, xCell, and quanTIseq algorithms. Blue represents negative Spearman correlation coefficients and red represents positive ones.

ESTIMATE, TIMER, xCell, and quanTIseq algorithms were used to infer the levels of infiltrating immune and stromal cells in tumor tissues and tumor purity. Higher immune scores were related to higher GBP1 expression in 32 cancer types except for THYM (Figure 2A). Both stromal and ESTIMATE scores were positively associated with GBP1 expression in 33 cancer types (Figure 2B, C). Immune infiltration levels of CD8^+^ T cells, B cells, neutrophils, macrophages, and myeloid dendritic cells were positively correlated with GBP1 expression in most cancer types, especially BLCA, CESC, COAD, HNSC, KICH, LUAD, LUSC, SKCM, and PRAD (Figure 2D, E). Evaluated by multiple algorithms, tumors with higher GBP1 expression had more infiltration by immune and stromal cells and lower tumor purity. The lower tumor purity that occurred in these tumors is probably because the immune cell infiltration was increased while the ratio of tumor cells was correspondingly decreased.

### GBP1 Alteration in Pan-Cancer

**FIGURE 3:**
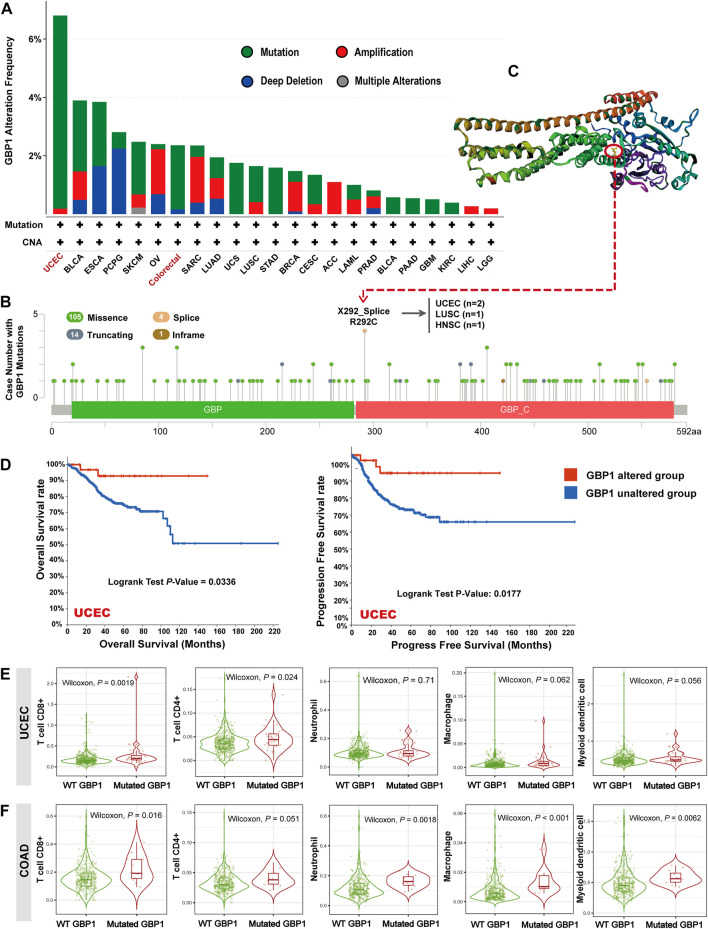
GBP1 alterations in different tumors of TCGA. **(A)** GBP1 alteration frequency with mutation types. **(B)** GBP1 mutation site. **(C)** The mutation site with the highest alteration frequency (X292_Splice/R292C) in the 3D structure of GBP1. **(D)** Kaplan–Meier curve for OS and PFS of UCEC patients with or without GBP1 alteration. The infiltration of CD8^+^ T cells, CD4^+^ T cells, neutrophils, macrophages, and myeloid dendritic cells between wild-type (WT) and mutated GBP1 groups of patients with UCEC **(E)** and COAD **(F)**.

The highest alteration frequency of GBP1 (>6%) appeared for patients with UCEC (Figure 3A). The “mutation” was the primary type of alteration in patients with UCEC and colorectal cancer (COAD and READ). The “deep deletion” type of CNA was the primary type in PCPG, and the “amplification” was the primary type in OV and SARC. As shown in Figure 3B, missense mutation was the main type of GBP1 mutation. The mutations (X292_splice and R292C) at site 292 of GBP1 protein were detected in two patients with UCEC, one patient with LUSC, and one patient with HNSC, which can induce GBP1 protein splicing and missense mutations. Then, we presented site 292 in the 3D structure of GBP1 protein (Figure 3C).

The GBP1-altered group of UCEC patients had better OS (*p* = 0.0336) and progress-free survival (PFS) (*p* = 0.0117) than the GBP1-unaltered group (Figure 3D). However, no significant difference in OS and PFS was detected in other cancer types, probably because of the low GBP1 alteration rates. Notably, patients with UCEC with mutated GBP1 showed more abundant CD4^+^ (*p* = 0.024) and CD8^+^ (*p* = 0.002) T cell infiltrations than those with wild-type (WT) GBP1 (Figure 3E). A similar pattern was observed in COAD, where GBP1 mutation was associated with more intense infiltrations of CD8^+^ T cells (*p* = 0.016), neutrophils (*p* = 0.002), macrophages (*p *< 0.001), and myeloid dendritic cells (*p* = 0.006) (Figure 3F). These results suggested that GBP1 mutation affected the survival prognosis of patients with UCEC *via* increasing the infiltration of immune cells in the tumor tissues.

### GBP1-Related Gene and Protein Enrichment Analysis

**FIGURE 4:**
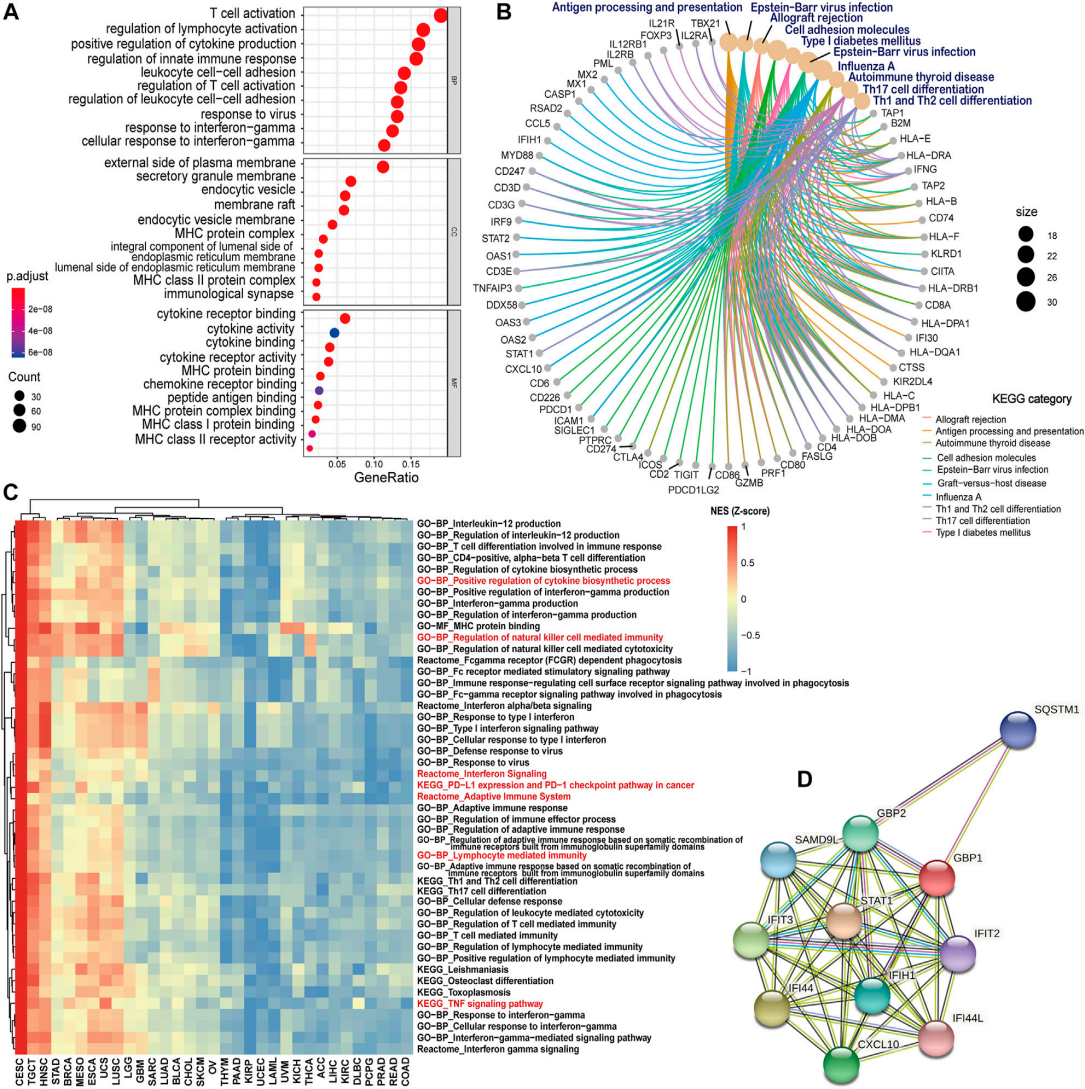
GBP1-related gene and protein enrichment analysis. **(A)** The top 10 GO enrichment significance terms of GBP1-related gens in three functional groups: biological processes (BP), cell composition (CC), and molecular function (MF). **(B)** KEGG pathway analysis of GBP1-related genes. **(C)** Heatmap comparison of significant GO terms, KEGG, and Reactome pathways across 33 cancer types. For each term and pathway, the normalized enrichment scores (NES) were normalized by *Z*-score. **(D)** PPI networks of GBP1 using the STRING tool. Each node represented all the proteins produced by a single, protein-coding gene locus, and each edge represented the predicted functional associations.

To explore the potential function of GBP1-related genes across pan-cancer patients, a total of 673 GBP1-related genes were selected for GO and KEGG analyses. The top GO terms included T cell activation, positive regulation of cytokine production, and cytokine activity (Figure 4A). KEGG pathway analysis also showed that GBP1-related genes were involved in antigen processing and presentation and Th1 and Th2 cell differentiation pathways (Figure 4B).

To compare the enrichment degree of GBP1-related terms and pathways in different cancer types, we performed GSEA for each cancer type. GBP1-related genes were more enriched in immune-related GO terms (lymphocyte-mediated immunity and regulation of natural killer cell-mediated immunity), KEGG pathways (PD-L1 expression and PD-1 checkpoint pathway in cancer and THF signaling pathway), and Reactome pathways (adaptive immune system and interferon signaling) for patients with CESC, TGCT, and HNSC compared to those with KIRP, UCEC, and LAML (Figure 4C).

Furthermore, the PPI network of GBP1, which consisted of 11 nodes and 47 edges, showed the GBP1-related proteins including STAT1, IFI44, IFIT3, CXCL10, GBP2, IFIH1, SAMD9L, SQSTM1, IFIT2, and IFI44L (Figure 4D).

### Analysis of GBP1 Expression and Immunotherapy

#### The Association of GBP1 Expression With the Expression of Immune Checkpoints and Tumor Mutational Burden

**FIGURE 5:**
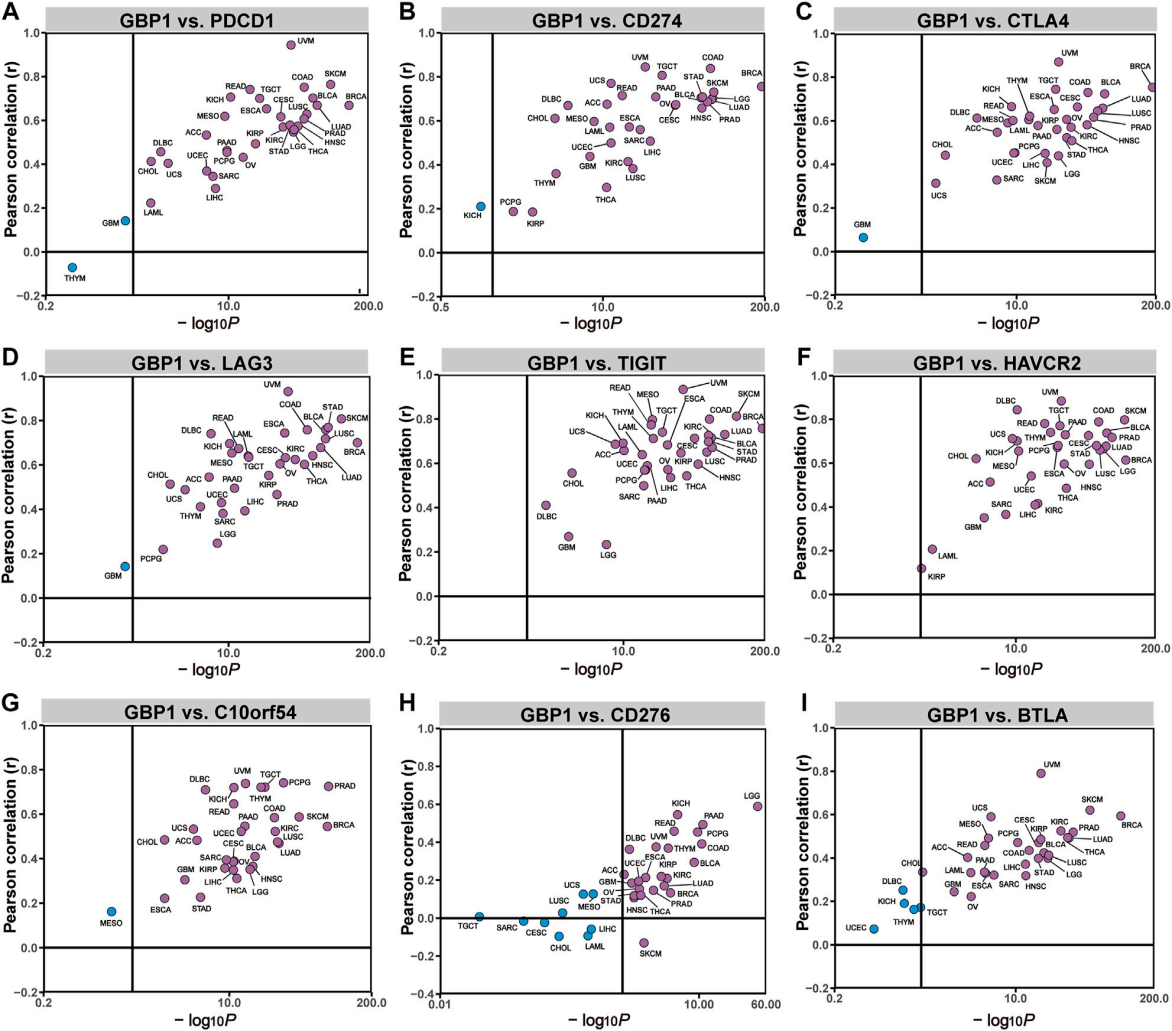
correlation between GBP1 expression and the expression levels of immune checkpoints. The correlation of GBP1 expression with PDCD1 **(A)**, CD274 **(B)**, CTLA4 **(C)**, LAG3 **(D)**, TIGIT **(E)**, HAVCR2 **(F)**, C10orf54 **(G)**, CD276 **(H)**, and BTLA **(I)** across 33 cancer types.

To determine the potential role of GBP1 expression in cancer immunotherapy, we investigated the correlation between GBP1 expression and some biomarkers such as immune checkpoints and TMB. In 33 cancer types, TIGIT and HAVCR2 expression had positive correlations with GBP1 expression (Figure 5E, F). PDCD1, CD274, CTLA4, LAG3, C10orf54, and BTLA expression had positive correlations in most cancer types (Figure 5A–D, G, I), and in approximately half of the cancer types, GBP1 expression is positively correlated with CD276 expression (Figure 5H). GBP1 expression was positively correlated with TMB of BRCA (*p* = 0.001), COAD (*p *< 0.001), LUAD (*p* = 0.004), LUSC (*p* = 0.012), rectum adenocarcinoma (READ) (*p* = 0.0058), STAD (*p *< 0.001), and THYM (*p* = 0.023) (Supplementary Figure S4).

#### The Association of GBP1 Expression With Anti-Tumor Immune Phenotype

**FIGURE 6:**
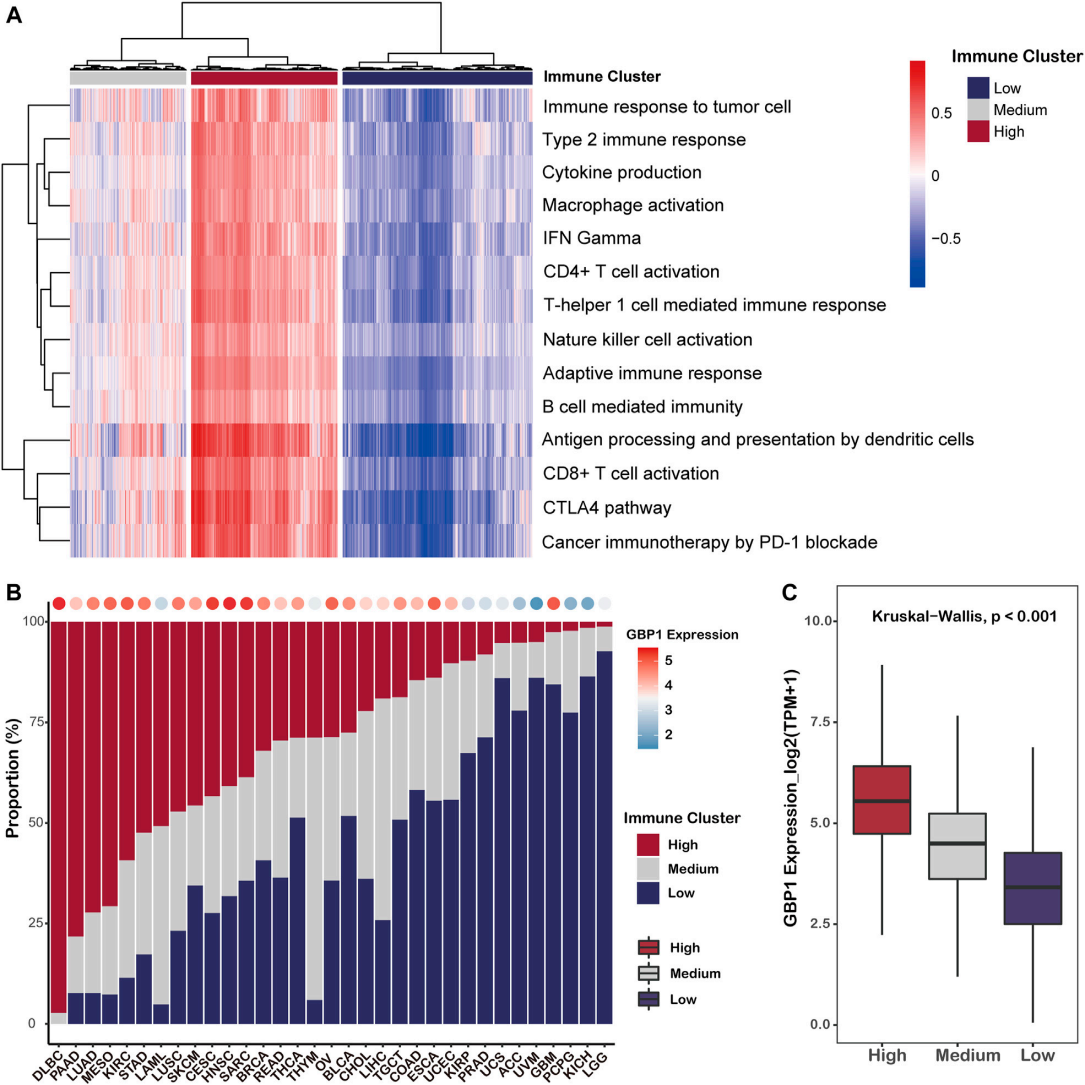
Clustering of pan-cancer patients based on their anti-tumor phenotypes. **(A)** Heatmap showing the pan-cancer patients grouped by hierarchical clustering using the GSVA enrichment scores for 14 immune-related gene sets. Patients were clustered in three major groups defined as low immune cluster (LIC), medium immune cluster (MIC), and high immune cluster (HIC). **(B)** Proportion of three immune clusters in each cancer type. Bubbles at the top of the graph represent the correlation of the proportion of HIC with average GBP1 expression in each cancer type. **(C)** Boxplot showing the expression level of GBP1 in each immune cluster.

Based on the anti-tumor immune phenotype, a clustering analysis separated pan-cancer patients into three immune clusters, including HIC (32% of pan-cancer patients), MIC (26%), and LIC (42%) (Figure 6A). The proportions of three immune clusters of 33 cancer types are shown in Figure 6B, and we found that the average expression level of GBP1 in each cancer type was positively correlated with the corresponding proportion of HIC (*R* = 0.58, *p *< 0.001) (Figure 6B; Supplementary Figure S5). In addition, patients belonging to HIC had significantly higher GBP1 expression than those belonging to MIC or LIC (*p *< 0.001) (Figure 6C). To exclude the possibility that this difference was driven by a few cancer types with numerous patients, we compared the GBP1 expression of three clusters in each cancer type (Supplementary Figures S6 and S7). Patients with high GBP1 expression were more likely to belong to HIC and displayed “hot” anti-tumor immune phenotypes.

#### Prediction of Immunotherapy Response in Patients With Different Expression Levels of GBP1

**FIGURE 7:**
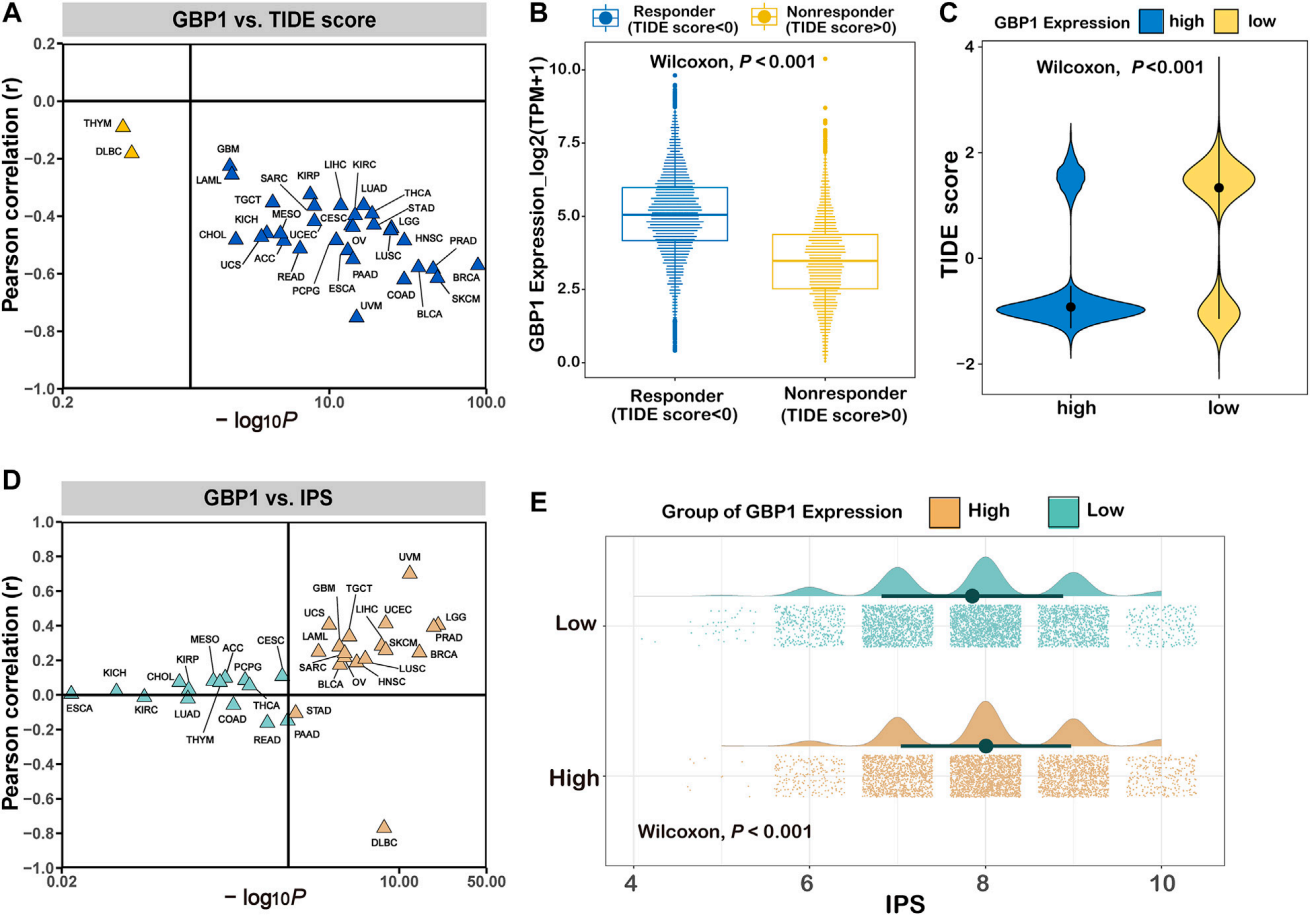
Prediction of immunotherapy response in patients with different expression levels of GBP1. **(A)** The correlation between GBP1 expression and TIDE scores across 33 cancer types. **(B)** The expression level of GBP1 in responders and non-responders. **(C)** The distribution of TIDE score in the high and low GBP1 expression groups. **(D)** The correlation between GBP1 expression and IPS across 33 cancer types. **(E)** The distribution of IPS in the high and low GBP1 expression groups. TIDE, immune dysfunction and exclusion; IPS, immunophenoscore.

To infer the efficacy of immunotherapy, we calculated TIDE scores and IPS for pan-cancer patients. GBP1 expression negatively correlated with TIDE scores in 31 cancer types apart from THYM and DLBC (Figure 7A). Responders to immunotherapy (TIDE score <0) had higher GBP1 expression than non-responders (TIDE score >0) (*p *< 0.001) (Figure 7B). Pan-cancer patients in the high GBP1 expression group had lower TIDE scores (*p *< 0.001) (Figure 7C). Similar differences were also found in 25 cancer types (Supplementary Figures S8 and S9). GBP1 expression positively correlated with IPS in 16 cancer types (Supplementary Figure S7D). The IPS in pan-cancer patients with high GBP1 expression was higher than those with low GBP1 expression (Figure 7E). Moreover, the difference of IPS between high and low GBP1 expression groups was significant in 17 cancer types (Supplementary Figures S10 and S11). Overall, patients with high GBP1 expression might show low tumor immune dysfunction and exclusion and high immunogenicity, resulting in better responses to immunotherapy.

#### Validation of the Predictive Capacity of GBP1 Expression in Immunotherapy

**FIGURE 8:**
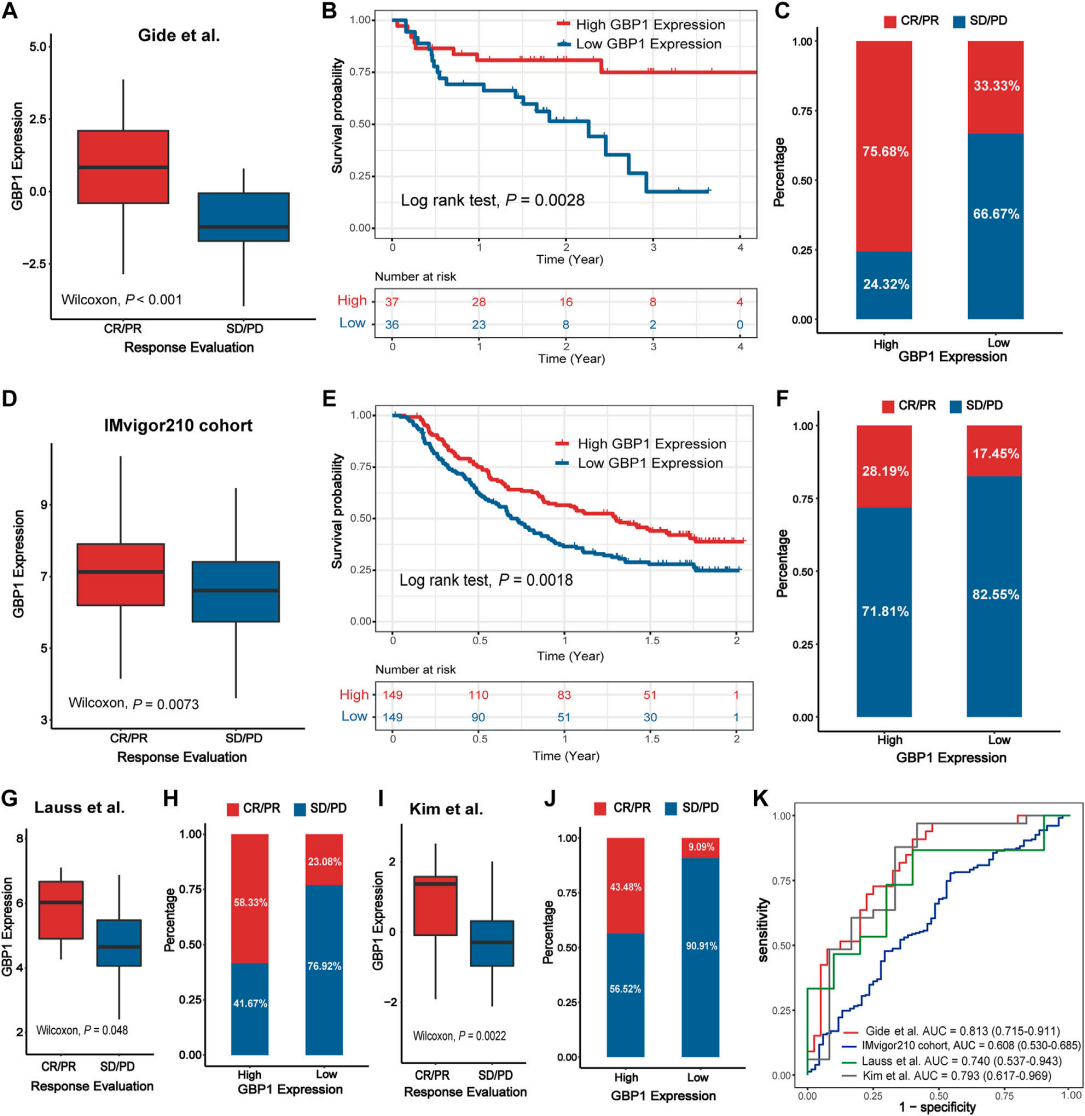
Validation of the predictive capacity of GBP1 expression in immunotherapy. The expression level of GBP1 in groups with a different immunotherapy response status in the Gide et al. cohort **(A)**, IMvigor210 cohort **(D)**, Lauss et al. cohort **(G)**, and Kim.et al. cohort **(I)**. Kaplan–Meier curves for patients with high and low GBP1 expression in the Gide et al. cohort **(B)** and IMvigor210 cohort **(E)**. The proportion of responders (CR/PR) and non-responders (SD/PD) in the high and low GBP1 expression groups in the Gide et al. cohort **(C)**, IMvigor210 cohort **(F)**, Lauss et al. cohort **(H)**, and Kim.et al. cohort **(J)**. Receiver operating characteristics (ROC) for GBP1 expression in the four immunotherapy cohorts **(K)**. AUCs with 95% confidence interval (CI) were provided.

Higher GBP1 expression was correlated with better clinical response to immunotherapy in the Gide et al. cohort (Wilcoxon test, *p *< 0.001) (Figure 8A). For the Gide et al. cohort, patients with high GBP1 expression had a longer OS than those with low GBP1 expression (log-rank test, *p* = 0.0028) (Figure 8B). The proportion of responders (CR/PR) was higher in the high-expression group than in the low-expression group (chi-square test, *p* < 0.001) (Figure 8C). Similar outcomes were observed in the IMvigor210 cohort (Wilcoxon test, *p* = 0.0073; log-rank test, *p* = 0.0018; chi-square test, *p* = 0.0272) (Figure 8D–F), the Lauss et al. cohort (Wilcoxon test, *p* = 0.0048; Fisher test, *p* = 0.1107) (Figure 8G, H), and the Kim et al. cohort (Wilcoxon test, *p* = 0.0022; chi-square test, *p* = 0.0091) (Figure 8I, J). After adjusting age, gender, TCGA subtype, TMB, and PD-L1 expression, GBP1 expression was identified as an independent risk factor for OS in the Gide et al. and IMvigor210 cohorts (Supplementary Figure S12A, C). However, there was no difference in OS between the high and low GBP1 expression groups due to the small sample size of the Lauss et al. cohort. Survival data were missing for the Kim et al. cohort.

To determine the predictive power of GBP1 expression for the proportion of responders, we performed an ROC validation. The AUC values were 0.813 (Gide et al. cohort), 0.607 (IMvigor210 cohort), 0.740 (Lauss et al. cohort), and 0.793 (Kim et al. cohort) (Figure 8K). Furthermore, the predictive powers of GBP1, PD-L1 expression, and TMB were similar in the Gide et al. cohort (GBP1 AUC: 0.813; PD-L1 AUC: 0.787) and IMvigor210 cohort (GBP1 AUC: 0.608; PD-L1 AUC: 0.604; TMB AUC: 0.645) (Supplementary Figure S12B, D).

## Discussion

GBP1 is a large gtpase of the dynamin superfamily governing cellular responses to infection, inflammation, and cancer (Honkala et al., 2019). Several studies have addressed the role of GBP1 in regulating immune response and repressing cell proliferation. GBP1-mediated actin cytoskeleton remodeling may contribute to regulating innate and adaptive immune defense (Ostler et al., 2014), and GBP1 can modify T cell activation *via* cytoskeleton-dependent cellular functions (Forster et al., 2014). The α9-helix of GBP1 mediated the anti-proliferative cell response to IFN-γ by inhibiting the Hippo signaling transcription factor TEAD (Unterer et al., 2018). However, a systematic analysis of GBP1 impacting clinical efficacy and tumor immune microenvironment changes in pan-cancer patients is still scarce. In this study, we comprehensively analyzed GBP1 in a total of 33 cancer types in TCGA and proposed GBP1 expression as a novel biomarker for immunotherapy response.

The pan-cancer analysis demonstrated that GBP1 was differentially expressed between tumors and normal tissues across many cancer types. GBP1 expression in KIRP, LGG, THYM, and UVM was related to poor prognosis but with better OS in OV and SKCM. Previous studies found the crucial role of GBP1 in tumor proliferation, metastasis, and treatment resistance (Britzen-Laurent et al., 2013; Mustafa et al., 2018; Song and Wei, 2020). GBP1 may restrain cancer cell proliferation as it inhibits endothelial and epithelial proliferation after cytokine stimulation (Honkala et al., 2019). Transcriptional and immunohistochemical profiling of patient samples has revealed that high GBP1 signatures were associated with tumor reduction in breast cancer and SKCM (Ascierto et al., 2012; Wang et al., 2018). However, GBP1 expression promoted tumor progression in oral cavity squamous cell carcinoma and ovarian cancer (Yu et al., 2011; Wadi et al., 2016). GBP1 expression was also correlated with metastasis in both lung and breast cancers, where lung adenocarcinoma cells and brain-metastasizing breast cancer cells showed decreased migration after silencing of GBP1 (Mustafa et al., 2018; Song and Wei, 2020). Furthermore, high GBP1 expression may also act as a mediator of paclitaxel resistance in human ovarian cancer cell lines (Wadi et al., 2016) and radioresistance in a variety of cancer cell lines (Fukumoto et al., 2014).

Tumor microenvironment (TME) consists of tumor cells and non-cancerous components such as immune cells, fibroblasts, endothelial cells, and extracellular matrix (Ochoa de Olza et al., 2020). A well-described biomarker of non-response to immunotherapy is the absence or low presence of lymphocytes in the TME, so-called cold tumors. In contrast, tumors with hot TME (hot tumors) can respond better to immunotherapy, leading to better tumor control and therapeutic outcomes (Ochoa de Olza et al., 2020). Our study first provided evidence of the correlation between GBP1 and hot TME in the pan-cancer context. Tumors with higher GBP1 expression had significantly lower tumor purity, more stromal cells, and infiltrating immune cells. It is noteworthy that most GBP1 protein expression was located in the cytoplasm and membrane of tumor cells rather than the stromal or immune cells, suggesting that the positive correlations between GBP1 expression and immune cell infiltration were not attributed to the increased numbers of the cell expressing GBP1. Also, GBP1 mutation facilitated the intratumoral immune cell infiltration, resulting in elevated anti-tumor responses and a better prognosis of UCEC patients.

Functional enrichment analysis indicated that GBP1-related genes were enriched in immune-related GO terms, KEGG, and Reactome pathways. GBP1-related genes included numerous IFN-stimulated genes (ISGs), for example, IRF9, STAT1/2, and OASs (Schneider et al., 2014). The clinical ICI response can be predicted by ISGs expressed by immune cells, especially ISGs typically associated with IFNG signaling. In contrast, ISGs expressed in cancer cells can predict resistance to ICIs (Benci et al., 2019). Moreover, other GBPs, the close relatives of GBP1, were reported to be associated with the malignancy of tumors and the prognosis of cancer patients. GBP2, GBP3, and GBP5 overexpression enhanced the invasion and migration of GBM cells *in vitro* and *in vivo* (Xu et al., 2018; Yu et al., 2020; Yu et al., 2021). GBP4 and other eight differentially expressed genes constituted an immune-relevant gene signature for predicting the prognosis of patients with muscle-invasive bladder cancer (MIBC) (Jiang et al., 2020). GBP5 was identified as a prognostic gene in the TME of hepatocellular carcinoma and gastrointestinal stromal tumors (Blakely et al., 2018; Xiang et al., 2021). Low GBP6 expression was correlated with poor cell differentiation and lymph node metastasis in tongue squamous cell carcinoma (TSCC), and low GBP7 expression was linked with short OS in HNSC patients (Liu et al., 2020; Wu et al., 2020). GBP5 and GBP6 were increased in cardiomyocytes of ICI-associated myocarditis (ICIM) patients compared to patients with dilated cardiomyopathy and virus-induced myocarditis (Finke et al., 2021). Current evidence does not show the association between the other GBPs and response to immunotherapy. Among the 10 GBP1-related proteins, IFIT3 is an interferon-induced protein, and high IFIT3 expression in hepatocellular carcinoma patients predicted a better response to IFN-α therapy (Yang et al., 2017). IFI44 expression was positively correlated with the infiltration of CD4^+^ T cells and macrophages as well as neutrophils in HNSC (Pan et al., 2020). STAT1 mediated cellular responses to cytokines and inhibited T cell exhaustion, which promoted anti-tumor immune responses in HNSC (Ryan et al., 2020). CXCL10 can induce monocyte and T-lymphocyte chemotaxis, leading to tumor suppression (Tokunaga et al., 2018). GBP2 was associated with a better prognosis in breast cancer and a more efficient T cell response (Godoy et al., 2014). Overall, GBP1 and its related proteins may elicit immune cell chemotaxis and infiltration while inhibiting T cell exhaustion.

Whereas, immune cell infiltration did not lead to good clinical benefit in overall patients with high GBP1 expression. It is potentially because of the elevated expression of immune checkpoints and activation of immunosuppressive pathways. Specifically, the infiltrating T cells displayed a dysfunctional phenotype characterized by high expression of PD-1, LAG3, and TIM3 (van der Leun et al., 2020). Another reason for the poor prognosis of patients with high GBP1 expression was the resistance to chemotherapy and radiotherapy, as patients recorded in the TCGA database tended to receive conventional chemotherapy and radiotherapy. Immunotherapy that relies on blocking immune checkpoints may be more effective in tumors with high GBP1 expression that contain more infiltrating immune cells and higher expression of immune checkpoints (Yoshihara et al., 2013). The analyses based on the four immunotherapy cohorts further validated this hypothesis.

Upregulation of immune checkpoint molecules is an important strategy to allow tumor cells to escape from anti-tumor immune attacks (Darvin et al., 2018). ICI therapy targets immune checkpoint molecules to reinvigorate anti-tumor immune responses. However, it remains a dilemma for identifying which patients will benefit from immunotherapy (Darvin et al., 2018). PD-L1 expression is now routinely used to determine whether to give immunotherapy in several cancer types (Davis and Patel, 2019). Another validated predictor is TMB, presumed into the production of neoantigens that can induce immunogenicity (Osipov et al., 2020). Patients with higher PD-L1 expression (tumor proportion score >50%) and TMB (≥10 mutations per megabase) had a better clinical response to ICI therapy without significant additional toxicity (Hui et al., 2017; Osipov et al., 2020). However, some responses occurred in PD-L1-negative tumors, while PD-L1-positive ones did not respond to immunotherapy due to the different localization of PD-L1 within the TME (Rizvi et al., 2015). The TMB threshold for clinical benefit needs determining because the threshold might differ by tumor type, testing platforms, and patient populations (Klempner et al., 2020). A six-gene IFN-γ signature (including IDO1, CXCL10, CXCL9, HLA-DRA, STAT1, and IFNG) was identified in a melanoma cohort of the KEYNOTE-001 study to predict response to pembrolizumab (Ribas et al., 2015).

This study found that GBP1 expression was positively associated with the expression of nine immune checkpoints in most cancer types. Abiko et al. (2015) demonstrated that IFN-γ, which induced GBP1 expression, promoted PD-L1 expression on ovarian cancer cells and mouse models. In addition, other immune checkpoint pathways such as CTLA-4 were reinforced by IFN-γ (Wang et al., 2001). Hence, we considered that IFN-γ signaling in tumors stimulated the expression of immune checkpoints, leading to the positive correlation between the expression of immune checkpoints and IFN-inducible GBP1. The subsequent analysis further demonstrated that patients with high GBP1 expression had hot anti-tumor immune phenotypes (HIC), low tumor immune dysfunction and exclusion (low TIDE scores), and high immunogenicity (high IPS). The validation in the immunotherapy cohorts showed that higher GBP1 expression indicated improved OS and better response, and GBP1 expression was identified as an independent risk factor for OS. Intriguingly, the association between PD-L1 expression and OS was not significant when both GBP1 and PD-L1 expressions were included in the multivariate Cox regression model, suggesting that PD-L1 expression was not an independent risk factor and GBP1 expression may have a greater impact on OS. In the context of an outpouring of novel ICIs, GBP1 expression holds promise to be a candidate biomarker for predicting the efficacy of immunotherapy, even for multiple ICI therapies.

In the present study, we demonstrated the predictive power of GBP1 expression of immunotherapy. However, we did not distinguish between immune monotherapy and combined immunotherapy. This study lacks investigation of the mechanism of GBP1 in acting directly within cancer cells or tumor microenvironment. Our next work will investigate if it is feasible to alter the tumor immune microenvironment by targeting GBP1.

In conclusion, our pan-cancer analysis of GBP1 indicated positive correlations between GBP1 and intratumoral immune infiltration, activation of immune-related pathways, and anti-tumor immune response in multiple cancer types. Furthermore, GBP1 expression can be a potential biomarker for immunotherapy response, facilitating the identification of suitable patients for tailoring optimal cancer therapeutic strategies.

## Data Availability

All of the data we used in this study were publicly available as described in the *Methods* section. Further inquiries can be directed to the corresponding authors.

## Glossary

ACC: Adrenocortical carcinoma
AUC: Area under the curve
B7-H3: B7 homolog 3 protein
BLCA: Bladder urothelial carcinoma
BRCA: Breast invasive carcinoma
BTLA: B and T cell lymphocyte attenuator
CESC: Cervical and endocervical cancers
CHOL: Cholangiocarcinoma
COAD: Colon adenocarcinoma
CNA: Copy number alteration
CPTAC: Clinical Proteomic Tumor Analysis Consortium
CTLA4: Cytotoxic T-lymphocyte-associated protein 4
DLBC: Diffuse large B-cell lymphoma
DFS: Disease-free survival
ESCA: Esophageal carcinoma
ESTIMATE: Estimation of STromal and Immune cells in MAlignant Tumor tissues using Expression data
FDR: False discovery rate
GBM: Glioblastoma multiforme
GBPs: Guanylate-binding proteins
GBP1: Guanylate-binding protein 1
GEPIA2: Gene Expression Profile Interactive Analysis, version 2
GO: Gene Ontology
GSEA: Gene Set Enrichment Analysis
GSVA: Gene Set Variation Analysis
GTPases: Guanosine triphosphatases
HIC: High immune cluster
HNSC: Head and neck squamous cell carcinoma
HPA: Human Protein Atlas
HR: Hazard ratios
ICIM: ICI-associated myocarditis
IFN: Interferon
IHC: Immunohistochemistry
ISG: IFN-stimulated gene
KEGG: Kyoto Encyclopedia of Genes and Genomes
KICH: Kidney chromophobe
KIRC: Kidney renal clear cell carcinoma
KIRP: Kidney renal papillary cell carcinoma
LAG3: Lymphocyte activation gene-3
LAML: Acute myeloid leukemia
LGG: Brain lower grade glioma
LIC: Low immune cluster
LIHC: Liver hepatocellular carcinoma
LUAD: Lung adenocarcinoma
LUSC: Lung squamous cell carcinoma
MESO: Mesothelioma
MIBC: Muscle-invasive bladder cancer
MIC: Medium immune cluster
MMP-1: Matrix metalloproteinase-1
NES: Normalized Enrichment Scores
NSCLC: Non-small cell lung cancer
OS: Overall survival
OV: Ovarian serous cystadenocarcinoma
PAAD: Pancreatic adenocarcinoma
PCPG: Pheochromocytoma and paraganglioma
PD-1: Programmed cell death 1
PD-L1: Programmed cell death ligand 1
PPI: Protein–protein interaction
PRAD: Prostate adenocarcinoma
READ: Rectum adenocarcinoma
RECIST: Response Evaluation Criteria in Solid Tumors
ROC: Receiver operating characteristic
SARC: Sarcoma
SKCM: Skin cutaneous melanoma
STAD: Stomach adenocarcinoma
STRING: Search Tool for the Retrieval of Interacting Genes
TGCT: Testicular germ cell tumors
THCA: Thyroid carcinoma
THYM: Thymoma
Th1: T helper type 1
TIGIT: T cell immunoglobulin and ITIM domain
TIM -3: Mucin-domain containing-3
TIMER2.0: Tumor Immune Estimation Resource, version 2.0
TMB: Tumor mutation burden
TPM: Transcripts per kilobase million
TSCC: Tongue squamous cell carcinoma
UCEC: Uterine corpus endometrial carcinoma
UCS: Uterine carcinosarcoma
UVM: Uveal melanoma
VISTA: V-domain immunoglobulin suppressor of T cell activation
3D: Three-dimensional
