# Supplementary material for: Guanylate-Binding Protein 1 as a Potential Predictor of Immunotherapy: A Pan-Cancer Analysis
Source: Front Genet. 2022 Feb 10;13:820135. doi: 10.3389/fgene.2022.820135 (PMC8867058; doi:10.3389/fgene.2022.820135)
Supplement: Supplementary file 2 [file Table1.DOCX]

**Table S1.** Direct links to the immunohistochemistry images of GBP1 protein from the HPA database.

| **Cancer type** | **Tumor** | **Normal** |
| --- | --- | --- |
| BLCA | https://www.proteinatlas.org/ENSG00000117228-GBP1/pathology/urothelial+cancer#img | https://www.proteinatlas.org/ENSG00000117228-GBP1/tissue/urinary+bladder#img |
| Breast Cancer | https://www.proteinatlas.org/ENSG00000117228-GBP1/pathology/breast+cance#img | https://www.proteinatlas.org/ENSG00000117228-GBP1/tissue/breast#img |
| CESC | https://www.proteinatlas.org/ENSG00000117228-GBP1/pathology/cervical+cancer#img | https://www.proteinatlas.org/ENSG00000117228-GBP1/tissue/cervix#img |
| COAD | https://www.proteinatlas.org/ENSG00000117228-GBP1/pathology/colorectal+cancer#img | https://www.proteinatlas.org/ENSG00000117228-GBP1/tissue/colon#img |
| CHOL | https://www.proteinatlas.org/ENSG00000117228-GBP1/pathology/liver+cancer#img | https://www.proteinatlas.org/ENSG00000117228-GBP1/tissue/liver#img |
| Lymphoma | https://www.proteinatlas.org/ENSG00000117228-GBP1/pathology/lymphoma#img | https://www.proteinatlas.org/ENSG00000117228-GBP1/tissue/lymph+node#img |
| HNSC | https://www.proteinatlas.org/ENSG00000117228-GBP1/pathology/head+and+neck+cancer#img | https://www.proteinatlas.org/ENSG00000117228-GBP1/tissue/nasopharynx#img |
| Renal Cancer | https://www.proteinatlas.org/ENSG00000117228-GBP1/pathology/renal+cancer#img | https://www.proteinatlas.org/ENSG00000117228-GBP1/tissue/kidney#img |
| Glioma | https://www.proteinatlas.org/ENSG00000117228-GBP1/pathology/glioma#img | https://www.proteinatlas.org/ENSG00000117228-GBP1/tissue/cerebral+cortex#img |
| LIHC | https://www.proteinatlas.org/ENSG00000117228-GBP1/pathology/liver+cancer#img | https://www.proteinatlas.org/ENSG00000117228-GBP1/tissue/liver#img |
| LUSC | https://www.proteinatlas.org/ENSG00000117228-GBP1/pathology/lung+cancer#img | https://www.proteinatlas.org/ENSG00000117228-GBP1/tissue/lung#img |
| OV | https://www.proteinatlas.org/ENSG00000117228-GBP1/pathology/ovarian+cancer#img | https://www.proteinatlas.org/ENSG00000117228-GBP1/tissue/ovary#img |
| PAAD | https://www.proteinatlas.org/ENSG00000117228-GBP1/pathology/pancreatic+cancer#img | https://www.proteinatlas.org/ENSG00000117228-GBP1/tissue/pancreas#img |
| STAD | https://www.proteinatlas.org/ENSG00000117228-GBP1/pathology/stomach+cancer#img | https://www.proteinatlas.org/ENSG00000117228-GBP1/tissue/stomach#img |
| THCA | https://www.proteinatlas.org/ENSG00000117228-GBP1/pathology/thyroid+cancer#img | https://www.proteinatlas.org/ENSG00000117228-GBP1/tissue/thyroid+gland#img |
