# Supplementary material for: Guanylate-Binding Protein 1 as a Potential Predictor of Immunotherapy: A Pan-Cancer Analysis
Source: Front Genet. 2022 Feb 10;13:820135. doi: 10.3389/fgene.2022.820135 (PMC8867058; doi:10.3389/fgene.2022.820135)
Supplement: Supplementary file 11 [file Table2.DOCX]

**Table S2.** Gene sets selected to make the cluster analysis.

| **Gene set** | **Molecular Signatures Database (MsigDB)** | **Brief description** |
| --- | --- | --- |
| Immune response to tumor cell | GOBP_IMMUNE_RESPONSE_TO_TUMOR_CELL | An immune system process that functions in the response of an organism to a tumor cell. |
| Type 2 immune response | GOBP_TYPE_2_IMMUNE_RESPONSE | An immune response which is associated with resistance to extracellular organisms, which is orchestrated by the production of particular cytokines, most notably IL-4, IL-5, IL-10, and IL-13, by any of a variety of cell types including T-helper 2 cells, eosinophils, basophils, mast cells, and nuocytes, resulting in enhanced production of certain antibody isotypes and other effects. |
| Cytokine production | GOBP_CYTOKINE_PRODUCTION_INVOLVED_IN_IMMUNE_RESPONSE | The appearance of a cytokine due to biosynthesis or secretion following a cellular stimulus contributing to an immune response, resulting in an increase in its intracellular or extracellular levels. |
| Macrophage activation | GOBP_MACROPHAGE_ACTIVATION | A change in morphology and behavior of a macrophage resulting from exposure to a cytokine, chemokine, cellular ligand, or soluble factor. |
| IFN Gamma | REACTOME_INTERFERON_GAMMA_SIGNALING | Interferon gamma signaling |
| CD4+ T cell activation | GOBP_CD4_POSITIVE_ALPHA_BETA_T_CELL_ACTIVATION | The change in morphology and behavior of a CD4-positive, alpha-beta T cell resulting from exposure to a mitogen, cytokine, chemokine, cellular ligand, or an antigen for which it is specific. |
| T-helper 1 cell mediated immune response | GOBP_T_HELPER_1_TYPE_IMMUNE_RESPONSE | An immune response which is associated with resistance to intracellular bacteria, fungi, and protozoa, and pathological conditions, and which is typically orchestrated by the production of particular cytokines by T-helper 1 cells, most notably interferon-gamma, IL-2, and lymphotoxin. |
| Nature killer cell activation | GOBP_NATURAL_KILLER_CELL_ACTIVATION | The change in morphology and behavior of a natural killer cell in response to a cytokine, chemokine, cellular ligand, or soluble factor. |
| Adaptive immune response | GOBP_ADAPTIVE_IMMUNE_RESPONSE | An immune response mediated by cells expressing specific receptors for antigen produced through a somatic diversification process, and allowing for an enhanced secondary response to subsequent exposures to the same antigen (immunological memory). |
| B cell mediated immunity | GOBP_B_CELL_MEDIATED_IMMUNITY | Any process involved with the carrying out of an immune response by a B cell. |
| Antigen processing and presentation by dendritic cells | GOBP_DENDRITIC_CELL_ANTIGEN_PROCESSING_AND_PRESENTATION | The process in which a dendritic cell expresses antigen (peptide or lipid) on its cell surface in association with an MHC protein complex. |
| CD8+ T cell activation | GOBP_CD8_POSITIVE_ALPHA_BETA_T_CELL_ACTIVATION | The change in morphology and behavior of a CD8-positive, alpha-beta T cell resulting from exposure to a mitogen, cytokine, chemokine, cellular ligand, or an antigen for which it is specific. |
| CTLA4 pathway | BIOCARTA_CTLA4_PATHWAY | The Co-Stimulatory Signal During T-cell Activation |
| Cancer immunotherapy by PD-1 blockade | WP_CANCER_IMMUNOTHERAPY_BY_PD1_BLOCKADE | Cancer immunotherapy by PD-1 blockade. |
